# Uptake of health checks by residents from the Danish social housing sector – a register-based cross-sectional study of patient characteristics in the ‘Your Life – Your Health’ program

**DOI:** 10.1186/s12889-018-5506-6

**Published:** 2018-05-02

**Authors:** Lars Bruun Larsen, Annelli Sandbaek, Janus Laust Thomsen, Anne-Louise Bjerregaard

**Affiliations:** 1 Department of Public Health, Research Unit of General Practice, University of Southern Denmark, Odense, Denmark; 2 Department of Public Health, Section of General Practice, Aarhus University, Aarhus, Denmark; 3 The Research Unit for General Practice, Aarhus University, Aarhus, Denmark; 4 Department of Clinical Medicine, Research Unit for General Practice, Aalborg University, Aalborg, Denmark

**Keywords:** Health check, Health examination, Attendance, Uptake, Community health services, Social deprivation, Socially deprived areas, Social housing sector

## Abstract

**Background:**

Poor uptake among socio-economically disadvantaged and susceptible populations is a well-known challenge of general health check interventions, and is widely cited as one of the reasons for the lack of population level effects seen in many studies. We report on patient characteristics among attendees and non-attendees of health checks made available to residents in the social housing sector of the municipality of Aarhus. We focus on this general population, as well as a particular sub-group living in an exceptionally deprived social housing area, and discuss the properties of intervention uptake that we need to be aware of to qualify and compare the effects of general versus targeted health checks in socially deprived areas.

**Methods:**

Cross-sectionally in a sample of 6650 residents of the Aarhus social housing sector who were invited for a health check in the first year of the ‘Your Life – Your Health’ program. The analyses consisted of 1) descriptive analysis of the characteristics of attenders/non-attenders, 2) unadjusted and adjusted Poisson regression to examine associations of patient characteristics and uptake of health checks, and 3) decision tree analyses (CHAID) to examine interaction and homogeneity in patient characteristics among attenders.

**Results:**

Of the overall population 30% attended. In a nested cohort of people residing in a particularly deprived social housing settlement, 25% attended. Further, in the overall population, we found an association between the likelihood of taking up a health check and age, sex, country of origin, educational attainment, cohabitation, occupational status, and past medical treatment. In the nested cohort the association between uptake and medical treatment was non-significant, while the association between uptake and occupation was limited to people who were employed. These results resonate with past evidence on health check attendance.

**Conclusions:**

Attendance in the ‘Your Life – Your Health’ program is higher among people of a higher socio-economic status. This should be taken into consideration when analysing and interpreting the overall study effects. Moreover, the results suggest that a targeted approach in the social housing sector could be more effective than a mass screening approach. However, more information is required to make such assertion definitive.

**Electronic supplementary material:**

The online version of this article (10.1186/s12889-018-5506-6) contains supplementary material, which is available to authorized users.

## Background

Poor uptake among socio-economically disadvantaged and susceptible populations is a well-known challenge of general health check interventions, and is widely cited as one of the reasons for the lack of population level effects seen in many studies [1–5].

Due to suboptimal care and under-diagnosing among the socio-economically disadvantaged populations targeted interventions aimed at high-risk groups may contribute to increased socio-economic equity in terms of cardiovascular morbidity and mortality [6].

Risk-scoring is a widely recommended approach to identify high-risk groups in the general population [7]. However, an evaluation of risk scores demonstrates that neglect of social deprivation as a risk factor exacerbates social inequality in health [8]. In the UK, the National Health Service (NHS) Health Check identifies high-risk groups based on a combination of medical health record information and an area based deprivation score, the Townsend Deprivation Index [9, 10]. In accordance with the aforementioned evaluation of risk-scores, a recent assessment of the first 4 years of the NHS Health Check service indicates higher uptake among low socio-economic status (SES) populations than other SES populations [11].

Comprehensive medical record information on social deprivation is mostly unavailable for population health purposes. An alternative to risk-scores as the one used in the UK may be to target high-risk groups by using an area-based approach alone. The potential impact of a targeted strategy aimed at socially deprived areas has been shown in a Scottish simulation study, comparing mass screening and targeted screening of residents in socially deprived areas [12]. Under the assumption of 1) equal uptake independent of the level of deprivation and 2) a higher proportion of people at risk the higher the level of deprivation, the simulation suggest that the number needed to screen (NNS) to identify one at-risk patient were 16 using mass screening and 6 using targeted screening in socially deprived areas.

In the present study, we report on patient characteristics among attendees and non-attendees of health checks made available only to residents in the social housing sector of the municipality of Aarhus as a novel area-based approach to target health checks that can serve as an alternative to a risk-score based approach when medical information is unavailable for population health purposes. We focus on this general population, as well as a particular sub-group living in an exceptionally deprived social housing area, and discuss the properties of intervention uptake that we need to be aware of to qualify and compare the effects of general versus targeted health checks in socially deprived areas.

## Method

This study uses a cross-sectional method to look at patient characteristics in a sample of 6650 residents of the Aarhus social housing sector who were invited for a health check in the first year of the ‘Your Life – Your Health’ program.

The ‘Your Life – Your Health’ program is a three-year prevention and health promotion initiative initiated and funded by the municipality of Aarhus. The applied research aims to evaluate the real-world effects of health checks and subsequent behaviour-change interventions, and/or medical treatment among people residing in the Danish social housing sector. The Danish social housing sector is a non-profit sector comprising apartment buildings for the purpose of providing affordable and decent housing to people in need. The social housing sector population is characterised by high rates of unemployment, with many receiving social welfare. Ethnic minorities are also overrepresented in this sector (Table 1).

**Table 1:** Socio-demographic profile of the population in the Municipality of Aarhus, the social housing sector in the municipality and in the socially deprived settlement of the satellite health centre (Information retrieved from the social registry of the Municipality of Aarhus)

(November, 2013)	Municipality of Aarhus (*N* = 330.000)	Social housing sector in the municipality of Aarhus (*N* = 75.500)	Deprived social housing settlement (*N* = 6650)
Unemployment rate^a,b^	17.0%	37.5%	52.4%
Social welfare recipients^b^	11.5%	27.7%	37.2%
Non-western origin^c^	10.7%	30.3%	79.7%

^a^Includes students

^b^age 18 to 64

^c^Total population

### Population and sampling

A total of 19.874 citizens, born between 1943 and 1968 (45 to 70 years of age at the time of invitation), were sampled to participate in the ‘Your Life – Your Health’ program during three consecutive years starting from September 2014. The study population consisted of a closed cohort of 6.650 participants pre-randomized to enter the program during the first year of the three-year program. The pre-randomization sampling was based on the unique Danish Civil Registration Number system, as well as on residency in the social housing sector in the municipality of Aarhus (330.000 inhabitants) [13]. Due to unforeseen delays in the implementation of the program, people allocated to the first year were invited for health checks from the beginning of September 2014 to the end of February 2016.

### Setting

The health checks took place at two municipal health centres: 1) a central health centre, and 2) a satellite health centre. Municipal health centres are administered and funded by the municipality and represent the principal arena for primary preventive services in the Danish health care sector. This includes lifestyle counselling and training, and secondary prevention in the form of patient education aimed at chronic disease prevention such as type-2 diabetes (T2DM), cardiovascular disease (CVD), chronic obstructive pulmonary disease (COPD), low back pain and stress. The municipal health centres are staffed by health professionals from various professions, including nurses, physiotherapists, dieticians, and occupational therapists.

The central health centre is located in the city centre of Aarhus, the second largest town in Denmark with an inner urban city population of approx. 270.000 people. The satellite health centre is located in a socially deprived area in the city of Aarhus with a total population of 6650 people (Table 1). All participants (*N* = 1407) who were invited to a health check at the satellite health centre resided in this particular socially deprived area. A few of these participants (*N* = 7), however, were examined at the central health centre due to patient delay between the invitation and the examination. All other participants were invited to a health check at the central health centre (*N* = 5243).

### Invitation

Invitations were sent out by mail to the participants on a random and on-going basis during the period from the beginning of September 2014 to the end of February 2016. The invitations included an information leaflet and a pre-booked date and time for the health check. The original Danish invitation was translated into English, Arabic, Turkish, and Somali, while the information leaflet was in Danish only.

The pre-booked date and time had to be confirmed by the invited person by text message, telephone, or on the project homepage within 7 days of receiving the invitation. Pre-booked appointments could be postponed (e.g. in case of pregnancy, planned surgery, illness, or if having had a recent health check at the general practitioner). People who did not respond to the invitation were sent a reminder 3 weeks after the initial invitation. No further action was taken to contact non-responders.

### Intervention

#### Self-reported questionnaire on health and health behavior

Prior to the health check, citizens were kindly asked to visit the project homepage and fill in a questionnaire on self-reported health (SF-12) and health behavior (physical activity, smoking, alcohol consumption, and diet). They were also asked to provide consent for their information to be used for research purposes related to the project, and to have the results from the questionnaire and health check forwarded to the their general practitioner (GP). The information from the questionnaire was readily available to the municipal health professional undertaking the health check and counselling.

The questionnaire and the consent form were only available in Danish. If the person did not speak or understand Danish she could fill in the questionnaire at the health centre with the help of the health professional undertaking the health check.

#### Health check

The health check consisted of a health counselling session and an examination, and lasted an average of 1 h. The time was equally spent for counselling and examination. The examination included measurements of blood pressure, HbA1c, cholesterols, and lipid levels, in addition to weight, height, and waist circumference. Furthermore, measures of fitness level and maximal oxygen consumption (based on a bike exercise test) were obtained. The native language of the health professionals performing the counselling and examination was Danish. Interpreters were not available, if needed the patient could bring a friend or family member.

At the end of the health check, the patient was given a written feedback report, summarizing the results from the questionnaire and the examination.

### Data analysis

Data from the entire cohort as well as data exclusively from the socially deprived area were analysed. No statistical inferences were made between the two cohorts. The analyses consisted of 1) descriptive analysis of the characteristics of attenders/non-attenders, 2) unadjusted and adjusted Poisson regression to examine associations of patient characteristics and uptake of health checks, and 3) decision tree analyses (CHAID) to examine interaction and homogeneity in patient characteristics among attenders [14].

#### Inclusion and exclusion criteria

Participants who fulfilled the following criteria were excluded from the analyses: Persons who had died before the scheduled health check, and persons who had moved from the municipality of Aarhus before the health check.

The only inclusion criterion of the nested cohort of residents from the socially deprived settlement was residency in the settlement at the time of invitation.

#### Causal model

A causal direct acyclic graph (DAG) model was developed to guide the adjustments for confounders in the Poisson regression models. We used guidelines from www.dagitty.net to inform the statistical analysis of the association between patient characteristics and uptake of health checks [15]. With attendance to health checks as the outcome variable we built the DAG model on the current evidence on the determinants of attendance to health checks and the model of health care use, proposed by Andersen [16]. The model states that the use of health care is determined by environmental factors surrounding the health care sector, the characteristics of the health care system, and the expected outcome of using health services. Also of relevance are the predisposing characteristics of the patient, enabling factors in his/her environment, as well as the patient’s general need for health care, and his/her health behavior.

The DAG is included in Additional file 1 as well as a review of the current evidence base on the determinants of attending health checks.

#### Variables

##### Outcome variable

Health check attendance was used as the main outcome variable. It was specified in terms of *attenders* (people who attended the health check) and *non-attenders* (people who did not attend the health check).

##### Exposure variables

Exposure variables included patient characteristics identified in the DAG as determinants of uptake of health checks, as well as age, sex, country of origin, education, occupation, income, medical treatment, frequent attendance at GP, preventive consultations at GP, partner in project. All variables except for partner in project were retrieved from Statistics Denmark and the Danish Health Data Authority, and linked with variables from the ‘Your Life – Your Health’ program using the unique Danish civil registration number [17–22].

Age was defined as age at date of invitation and categorized in five-year age intervals. Date of invitation was missing for 363 people. Missing values were assigned the age of the person at the first of June 2015 as this date is the median date in the study period. No other assignments were made.

Educational level was defined as the highest degree of formal education completed by the first of October 2014. Educational level was classified according to the UNESCO categories and categorized into three groups: ≤10 years of education, 11–15 years of education, and > 15 years of education [23].

Occupation was defined as the occupational status ultimo November 2013. Occupation was categorized into five groups: being employed, self-employed, unemployed/on benefits, social welfare recipients or other. Unemployment benefits are for people who have been unemployed for less than 2 years and who are member of a voluntary unemployment benefit fund. Social welfare recipients are for unemployed people who are not member of an unemployment benefit fund or have been unemployed for more than 2 years. Basic social welfare represents 60% of the value of basic unemployment benefits. Others include, amongst others, a non-working person from a family that rely on one income only.

OECD-adjusted income level was defined by the household’s mean annual net income for the period 2011–2013, and adjusting this figure for family size, where the first adult would count for one, other adults and children above 14 years of age would count for 0.5, and children below 14 years of age would count for 0.3 [24]. In the absence of national poverty levels family’s available income was categorized into quartiles; Low (below 50% of median), middle-low, middle-high and high (50% above median).

Cohabitation data was retrieved from Statistics Denmark for the year 2015 and categorized in terms of *cohabiting* or *single*. Cohabitation was defined as two adults residing at the same address and who were either married, in a registered partnership, living together with at least one common child, or were born within 15 years of one another.

Information about participants’ country of origin was retrieved from Statistics Denmark for the year 2014 in order to reduce the amount of missing data due to people moving out of the specified social housing area. Country of origin was further categorized into three groups: western immigrants (person emigrated from a western country), non-western immigrants (person emigrated from a non-western country), or Danish (rest of the population).

We used participant’s use of particular prescription medication as an indicator of received medical treatment. Specifically, participants were categorised as receiving, or as having received medical treatment if they had been prescribed drugs included in the Anatomical Therapeutic Chemical (ATC) Classification System codes of C0 (cardiovascular system), A10 (drugs used in diabetes), R03 (drugs for obstructive airways diseases), N05A (antipsychotics), N05B (anxiolytics) and N06A (antidepressants) from Danish pharmacies during the period of January 2012 through to end of August 2014. Medical treatment was coded as a binary yes/no variable.

The frequency with which participants saw their GP was determined by the mean number of kept face-to-face appointments (administrative code 0101) and telephone consultations (administrative code 0201) with the GP during daytime hours between 2011 and 2013. Attenders were categorized as *frequent attenders* and *others*, where the former category represented the top 10% of people who were in touch with their GP the most [25, 26].

Participants, who had attended preventive consultations (administrative code 0120) at the GP during daytime hours in the two consecutive calendar years 2012 and 2013, were coded as a binary yes/no variable. The administrative code for preventive consultations includes counselling on both primary and secondary prevention and is thus used for both people at risk and people already in treatment.

*Partner in project* describes whether your partner, if cohabiting, attended a health check or not. Partner in project is categorised as a Yes/No and was derived from project data.

#### Confounding variables

A neighbourhood social deprivation score was calculated from a score developed by Bender et al. to examine the association between neighbourhood social deprivation and participation in general health checks [27]. The score is based on the proportion of people with basic education, rates of unemployment, and the proportion of people in the lowest income quartile in Denmark and categorized into quartiles high/middle high/middle low/low neighbourhood social deprivation. Information for the score was retrieved from the nationwide Danish social registries on health and social issues for all census districts in Denmark. In this way we could generate accurate local deprivation scores that reflect the relative social deprivation of the individual census districts in the municipality of Aarhus and nationally [17].

#### Statistical analysis

Characteristics of attenders and non-attenders are presented and compared using the chi-square test for comparison of proportions. Associations are presented as crude estimates for all exposure variables as well as estimates adjusted for age and sex and multivariable analyses minimally adjusted according to the DAG causal model. All estimates were 372 analysed using Poisson regression. For interpretative purposes Poisson regression and incidence rate ratios (IRR) was chosen over logistic regression and odds ratios (OR). The dependent variable attendance is hence understood as a continuous variable with counts being 0 or 1.

The Poisson regression was performed with robust error variance and presented with p-values and 95% confidence intervals (CI) of the IRR. Estimates are presented for the entire cohort of residents of the social housing sector, and for the nested cohort of residents of the socially deprived settlement.

A chi-squared automatic interaction detection (CHAID) decision tree analysis was performed to detect interaction between exposure variables, and to divide data into more homogenous groups [14]. The CHAID analysis groups variables into mutually exclusive subsets based on homogeneity through a series of chi-square tests. The grouping eventually ends up in a hierarchical order of optimal splits of nodes with the most explanatory exposure variable ranked at the top of the hierarchy. Patient characteristics and neighbourhood deprivation were used as categorical input for the CHAID analysis. The CHAID analysis was run with parent nodes defined at a minimum of 200 persons, child nodes defined at a minimum of 20 persons, and significance (α_merge_, α_split_, and *P*-value) set at ≤0.05.

Statistical analyses were performed using Stata14.

## Results

The study population consisted of 6650 persons. As one person died before the health check the statistical analyses were performed with *N* = 6649 persons. The attendance rate was 30% in the entire cohort and 25% in the nested cohort (Table 2). People with non-Danish origin constituted 30% of the entire cohort and originating from 92 countries, and 57% of the nested cohort originating from 53 countries. Social welfare recipients represented 53 and 63% of the overall and the nested cohorts, respectively. About half lived alone and 60% received medical treatment. In the nested cohort 37% had less than 10 years of formal education while 51% belonged to the lowest income quartile.

**Table 2:** Crude estimates of the association between an exposure and attendance to the health check in the entire cohort and the nested cohort of residents invited to the satellite health centre

	Entire cohort				Nested cohort			
	Attendance	Non-attendance	Total	Missings	Attendance	Non-attendance	Total	Missings
n (pct)	1958 (29.5)	4691 (70.6)	6649 (100)	0 / 6649	355 (25.2)	1052 (74.8)	1407 (100)	0 / 1407
Age at first invitation				0 / 6649				0 / 1407
-49	332 (17.0)	1039 (22.2)	1371 (20.6)		60 (16.9)	268 (25.5)	328 (23.3)	
50–54	442 (22.6)	1065 (22.7)	1507 (22.7)		90 (25.4)	237 (22.5)	327 (23.2)	
55–59	413 (21.1)	963 (20.5)	1376 (20.7)		71 (20.0)	200 (19.0)	271 (19.3)	
60–64	367 (18.7)	766 (16.3)	1133 (17.0)		62 (17.5)	169 (16.1)	231 (16.4)	
65+	404 (20.6)	858 (18.3)	1262 (19.0)		72 (20.3)	178 (16.9)	250 (17.8)	
Sex				0 / 6649				0 / 1407
Female	1107 (56.5)	2397 (51.1)	3504 (52.7)		195 (54.9)	466 (44.3)	661 (47.0)	
Male	851 (43.5)	2294 (48.9)	3145 (51.9)		160 (45.1)	586 (55.7)	746 (53.0)	
Education (years)				432 / 6649				180 / 1407
< =10	479 (25.8)	1575 (36.1)	2054 (33.0)		89 (28.2)	361 (39.6)	450 (36.7)	
10–15	832 (44.8)	1822 (41.8)	2654 (42.7)		140 (44.3)	371 (40.7)	511 (41.7)	
> 15	547 (29.4)	962 (22.1)	1509 (24.3)		87 (27.5)	179 (19.7)	266 (21.7)	
Income (1000 dkk)				6 / 6649				3 / 1407
Low quartile (0-)	581 (29.7)	1734 (37.0)	2315 (34.9)		157 (44.2)	556 (53.0)	713 (50.8)	
Middle low quartile (161-)	756 (38.6)	1836 (39.2)	2592 (39.0)		108 (30.4)	355 (33.8)	463 (33.0)	
Middle high quartile (216-)	437 (22.3)	815 (17.4)	1252 (18.9)		68 (19.2)	116 (11.1)	184 (13.1)	
High quartile (283-)	184 (9.40)	300 (6.40)	484 (7.29)		22 (6.20)	22 (2.10)	44 (3.13)	
Occupational status				5 / 6649				3 / 1407
Employed	870 (44.4)	1616 (34.5)	2486 (37.4)		133 (37.5)	251 (23.9)	384 (27.4)	
Self-employed	43 (2.20)	78 (1.66)	121 (1.82)		6 (1.69)	22 (2.10)	28 (2.00)	
Unemployed/benefits	84 (4.29)	191 (4.08)	275 (4.14)		18 (5.07)	41 (3.91)	59 (4.20)	
Social welfare recipients	898 (45.9)	2639 (56.3)	3537 (53.2)		183 (51.5)	701 (66.8)	884 (63.0)	
Others	63 (3.22)	162 (3.46)	225 (3.39)		15 (4.23)	34 (3.24)	49 (3.49)	
Country of origin				0 / 6649				0 / 1407
Denmark	1429 (73.0)	3227 (68.8)	4656 (70.0)		176 (49.6)	430 (40.9)	606 (43.1)	
Western	107 (5.46)	185 (3.94)	292 (4.39)		23 (6.48)	47 (4.47)	70 (4.98)	
Non-western	422 (21.6)	1279 (27.3)	1701 (21.1)		156 (43.9)	575 (54.7)	731 (52.0)	
Cohabitation				8 / 6649				3 / 1407
Single	1049 (53.6)	2626 (56.1)	3675 (55.3)		151 (42.5)	510 (48.6)	661 (47.1)	
Cohabiting	908 (46.4)	2058 (43.9)	2966 (44.7)		204 (57.5)	539 (51.4)	743 (52.9)	
Partner in project				8 / 6649				3 / 1407
Yes	729 (37.3)	1641 (35.0)	2370 (35.7)		150 (42.3)	393 (37.5)	543 (38.7)	
No	1228 (62.8)	3043 (65.0)	4271 (64.3)		205 (57.8)	656 (62.5)	861 (61.3)	
Neighbourhood deprivation				7 / 6649				4 / 1407
Low deprivation	262 (13.4)	600 (12.8)	862 (13.0)		14 (3.94)	45 (4.29)	59 (4.21)	
Low middle deprivation	423 (21.6)	988 (21.1)	1411 (21.2)		76 (21.4)	174 (16.6)	250 (17.8)	
High middle deprivation	655 (33.5)	1389 (29.7)	2044 (30.8)		71 (20.0)	156 (14.9)	227 (16.2)	
High deprivation	618 (31.6)	1707 (36.4)	2325 (35.0)		194 (54.7)	673 (64.2)	867 (61.8)	
Medical treatment				0 / 6649				0 / 1407
Yes	1118 (57.1)	2934 (62.6)	4052 (60.5)		209 (58.9)	644 (61.2)	853 (61.3)	
No	840 (42.9)	1757 (37.5)	2597 (39.1)		146 (41.1)	408 (38.8)	554 (39.4)	
Frequent attenders to GP (top 10%)				0 / 6649				0 / 1407
Yes	149 (7.61)	415 (8.85)	564 (8.48)		29 (8.17)	81 (7.70)	110 (7.82)	
No	1809 (92.4)	4276 (91.2)	6085 (91.5)		326 (91.8)	971 (92.3)	1297 (92.2)	
Preventive consultation at GP				0 / 6649				0 / 1407
Yes	506 (25.8)	1271 (27.1)	1777 (26.7)		95 (26.8)	278 (26.4)	373 (26.5)	
No	1452 (74.2)	3420 (72.9)	4872 (73.3)		260 (73.2)	774 (73.6)	1034 (73.5)	

### Uptake in the entire cohort

Uptake in the entire cohort increased with age, was higher among women and people of Danish or western origin compared to people of non-western origin (Table 3). Adjusted estimates showed an association between uptake and increasing educational level, occupational status, cohabiting and not receiving medical treatment. Income, frequency of attendance at the GP, being registered with a preventive consultation at the GP, or whether your partner had also been invited to participate showed no impact on uptake (Table 3).

**Table 3:** Adjusted estimates of the association between an exposure and attendance to the health check in the entire cohort

	Model 1 (crude)		Model 2 (adjusted for age and sex)		Model 3 (minimal adjustment)	
	IRR (95% CI)	*p*-value		*p*-value	IRR (95% CI)	*p*-value
Age^a^						
-49	1 (−; −)		1 (−; −)		1 (−; −)	
50–54	1.21 (1.07;1.37)	0.002	1.21 (1.07;1.37)	0.002	1.21 (1.07;1.37)	0.002
55–59	1.24 (1.10;1.40)	0.001	1.24 (1.10;1.41)	0.001	1.24 (1.10;1.40)	0.001
60–64	1.34 (1.18;1.52)	0.000	1.34 (1.18;1.52)	0.000	1.34 (1.18;1.52)	0.000
65+	1.32 (1.17;1.50)	0.000	1.32 (1.17;1.49)	0.000	1.32 (1.17;1.50)	0.000
Sex^a^						
Female	1 (−; −)		1 (−; −)		1 (−; −)	
Male	0.86 (0.80;0.92)	0.000	0.86 (0.79;0.92)	0.000	0.86 (0.79;0.92)	0.000
Country of Origen^a^						
Denmark	1 (−; −)		1 (−; −)		1 (−; −)	
Western	1.20 (1.02;1.40)	0.027	1.21 (1.03;1.41)	0.020	1.19 (1.02;1.40)	0.027
Non-western	0.81(0.74;0.89)	0.000	0.85 (0.77;0.93)	0.001	0.81 (0.74;0.89)	0.000
Education^b^						
< =10	1 (−; −)		1 (−; −)		1 (−; −)	
10–15	1.34 (1.22;1.48)	0.000	1.37 (1.25;1.51)	0.000	1.36 (1.24;1.50)	0.000
> 15	1.56 (1.40;1.72)	0.000	1.57 (1.42;1.74)	0.000	1.56 (1.41;1.73)	0.000
Occupational status^c^						
Employed	1.38 (1.28;1.49)	0.000	1.51 (1.39;1.64)	0.000	1.38 (1.26;1.51)	0.000
Self-employed	1.40 (1.10;1.79)	0.008	1.59 (1.25;2.03)	0.000	1.46 (1.14;1.87)	0.003
Unemployed/benefits	1.20 (1.00;1.45)	0.053	1.38 (1.14;1.67)	0.001	1.27 (1.04;1.54)	0.018
Social welfare recipients	1 (−; −)		1 (−; −)		1 (−; −)	
Others	1.10 (0.89;1.37)	0.377	1.80 (1.14;2.85)	0.012	1.76 (1.09;2.83)	0.021
Income^d^						
Low quartile	1 (−; −)		1 (−; −)		1 (−; −)	
Middle low quartile	1.16 (1.06;1.28)	0.001	1.13 (1.03;1.24)	0.012	1.05 (0.95;1.17)	0.339
Middle high quartile	1.39 (1.25;1.54)	0.000	1.37 (1.23;1.52)	0.000	1.10 (0.97;1.25)	0.126
High quartile	1.52 (1.33;1.73)	0.000	1.47 (1.29;1.69)	0.000	1.14 (0.97;1.33)	0.108
Cohabitation^c^						
Single	1 (−; −)		1 (−; −)		1 (−; −)	
Cohabiting	1.07 (1.00;1.16)	0.066	1.09 (1.01;1.17)	0.023	1.11 (1.03;1.20)	0.007
Medical treatment^e^						
Yes	1 (−; −)		1 (−; −)		1 (−; −)	
No	1.17 (1.09;1.26)	0.000	1.23 (1.14;1.33)	0.000	1.22 (1.13;1.32)	0.000
Frequent attendance^f^						
Yes	1 (−; −)		1 (−; −)		1 (−; −)	
No	1.13 (0.98;1.30)	0.106	1.17 (1.01;1.35)	0.035	0.98 (0.84;1.13)	0.753
Preventive consultation^g^						
Yes	1 (−; −)		1 (−; −)		1 (−; −)	
No	1.05 (0.96; 1.14)	0.295	1.10 (1.00;1.20)	0.040	1.00 (0.91;1.10)	0.994
Partner in project^h^						
Yes	1 (−; −)		1 (−; −)		1 (−; −)	
No	0.94 (0.87;1.01)	0.084	0.94 (0.87;1.01)	0.096	0.98 (0.85;1.12)	0.732

Model 3 adjustments

^a^No adjustments

^b^Age, sex, country of origin

^c^Age, sex, country of origin, education

^d^Age, sex, country of origin, education, occupation

^e^Age

^f^Age, sex, education, occupation, cohabitation

^g^Age, sex, medical treatment, education, country of origin, frequent attendance, income, neighbourhood social deprivation, occupation, cohabitation

^h^Cohabitation

**Fig. 1:**
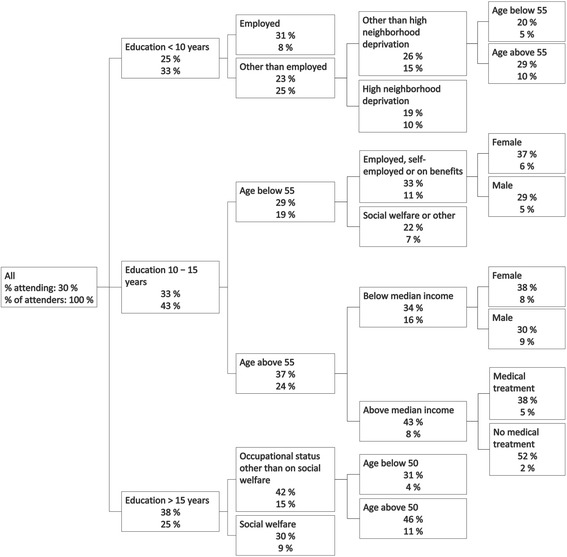
Decision tree plot of the entire cohort

The CHAID analysis of the entire cohort shows that education was the strongest predictor of attendance followed by age, and occupational status (Fig. 1). The CHAID also indicate large differences in the absolute uptake. A mere 19% of people with less than 10 years of education, who live in neighbourhoods with high deprivation, and who are not employed attended the health checks. By contrast, the uptake was as high as 52% among people over 55 years of age with 11 to 15 years of education, an income above the median, and not in treatment. Among people with less than 10 years of education the uptake among self-employed people was significantly higher than all other occupational categories. Among people with more than 15 years of education the uptake among social welfare recipients was lower than other occupational categories.

### Uptake in the nested cohort

The nested cohort likewise showed higher uptake among women and people of Danish and western origin, though age only differed slightly due to a lower uptake among people under the age of 50 (Table 4). Further, the adjusted analysis shows that people with higher education, who are cohabiting (as opposed to living along), and are being employed rather than unemployed is associated with higher uptake (Table 4). Unlike the analysis of the entire cohort, the analysis of the nested cohort suggests that medical treatment has no impact on uptake. Similarly, income, frequent attendance to GP, registration of a preventive consultation at the GP, and whether your partner was invited to participate indicate no impact on the uptake of health checks.

**Table 4:** Adjusted estimates of the association between an exposure and attendance to the health check in the nested cohort of residents invited to the satellite health centre

	Model 1 (crude)		Model 2 (adjusted for age and sex)		Model 3 (minimal adjustment)	
	IRR (95% CI)	*p*-value	IRR (95% CI)	*p*-value	IRR (95% CI)	*p*-value
Age^a^						
-49	1 (−; −)		1 (−; −)		1 (−; −)	
50–54	1.51 (1.13;2.01)	0.006	1.51 (1.13;2.01)	0.005	1.51 (1.13;2.01)	0.006
55–59	1.43 (1.06;1.94)	0.020	1.44 (1.06;1.94)	0.019	1.43 (1.06;1.94)	0.020
60–64	1.47 (1.07;2.01)	0.016	1.49 (1.09;2.03)	0.012	1.47 (1.07;2.01)	0.016
65+	1.57 (1.17;2.13)	0.003	1.56 (1.15;2.11)	0.004	1.57 (1.17;2.13)	0.003
Sex^a^						
Female	1 (−; −)		1 (−; −)		1 (−; −)	
Male	0.73 (0.61;0.87)	0.001	0.73 (0.61;0.87)	0.001	0.73 (0.61;0.87)	0.001
Country of Origen^a^						
Denmark	1 (−; −)		1 (−; −)		1 (−; −)	
Western	1.13 (0.79;1.62)	0.499	1.14 (0.80;1.64)	0.466	1.13 (0.79;1.62)	0.499
Non-western	0.74 (0.61;0.89)	0.001	0.77 (0.64;0.93)	0.007	0.74 (0.61;0.89)	0.001
Education^b^						
< =10	1 (−; −)		1 (−; −)		1 (−; −)	
10–15	1.39 (1.10;1.75)	0.006	1.47 (1.16;1.85)	0.001	1.45 (1.15;1.83)	0.002
> 15	1.65 (1.28;2.13)	0.000	1.70 (1.32;2.19)	0.000	1.67 (1.30;2.15)	0.000
Occupational status^c^						
Employed	1.67 (1.39;2.02)	0.000	1.83 (1.51;2.22)	0.000	1.67 (1.33;2.09)	0.000
Self-employed	1.04 (0.50;2.13)	0.925	1.32 (0.65;2.68)	0.444	1.12 (0.53;2.36)	0.766
Unemployed/benefits	1.47 (0.98;2.12)	0.061	1.66 (1.09;2.53)	0.019	1.33 (0.82;2.15)	0.247
Social welfare recipients	1 (−; −)		1 (−; −)		1 (−; −)	
Others	1.48 (0.95;2.30)	0.082	1.69 (1.10;2.60)	0.017	1.65 (1.04;2.63)	0.035
Income^d^						
Low quartile	1 (−; −)		1 (−; −)		1 (−; −)	
Middle low quartile	1.06 (0.85;1.31)	0.600	1.01 (0.81;1.25)	0.959	0.87 (0.67;1.11)	0.262
Middle high quartile	1.68 (1.33;2.12)	0.000	1.62 (1.29;2.05)	0.000	1.12 (0.82;1.52)	0.494
High quartile	2.27 (1.64;3.15)	0.000	2.11 (1.51;2.95)	0.000	1.41 (0.94;2.10)	0.095
Cohabitation^c^						
Single	1 (−; −)		1 (−; −)		1 (−; −)	
Cohabiting	1.20 (1.00;1.44)	0.048	1.25 (1.04;1.49)	0.017	1.30 (1.07;1.57)	0.007
Medical treatment^e^						
Yes	1 (−; −)		1 (−; −)		1 (−; −)	
No	1.08 (0.90;1.29)	0.434	1.16 (0.96;1.39)	0.121	1.20 (0.99;1.45)	0.069
Frequent attendance^f^						
Yes	1 (−; −)		1 (−; −)		1 (−; −)	
No	0.95 (0.69;1.32)	0.774	1.04 (0.75;1.44)	0.829	0.75 (0.53;1.06)	0.106
Preventive consultation^g^						
Yes	1 (−; −)		1 (−; −)		1 (−; −)	
No	0.99 (0.81;1.21)	0.902	1.06 (0.87;1.31)	0.556	0.98 (0.79;1.22)	0.885
Partner in project^h^						
Yes	1 (−; −)		1 (−; −)		1 (−; −)	
No	0.86 (0.72;1.03)	0.108	0.89 (0.74;1.06)	0.192	0.98 (0.75;1.28)	0.866

Model 3 adjustments

^a^No adjustments

^b^Age, sex, country of origin

^c^Age, sex, country of origin, education

^d^Age, sex, country of origin, education, occupation

^e^Age

^f^Age, sex, education, occupation, cohabitation

^g^Age, sex, medical treatment, education, country of origin, frequent attendance, income, neighbourhood social deprivation, occupation, cohabitation

^h^Cohabitation

**Fig. 2:**
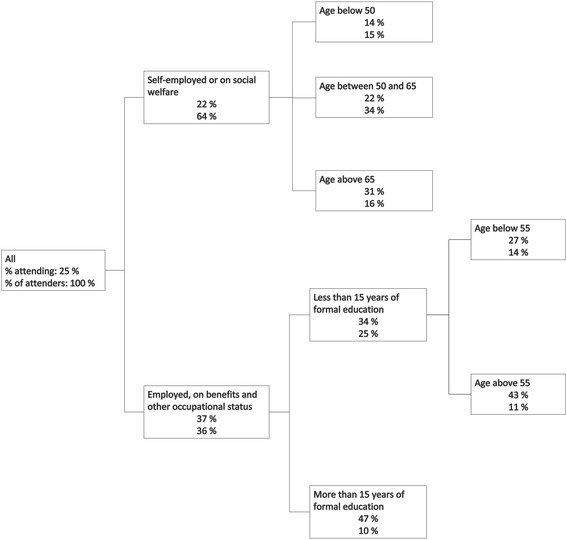
Decision tree plot of the nested cohort

The CHAID analysis show that occupation was the strongest predictor of attendance to the ‘Your Live – Your Health’ program in the nested cohort. Self-employed are grouped with those receiving social welfare, and employed with their unemployed counterparts who are on benefits. The results indicate lower attendance among self-employed and social welfare recipients (Fig. 2).

## Discussion

In this study on the patient characteristics of attendees and non-attendees at social housing sector health checks 30% of the overall population attended scheduled health checks. In a nested cohort of people residing in a particularly deprived social housing settlement, 25% attended. Comparable differences in uptake were seen in a Dutch study on health checks inviting an underserved population using postal invitations [28]. Further, in the overall population, we found an association between the likelihood of taking up an offer of a health check and age, sex, country of origin, educational attainment, cohabitation, occupational status, and past medical treatment. In the nested cohort the association between uptake and medical treatment was non-significant, while the association between uptake and occupation was limited to people who were employed. These results resonate with past evidence on health check attendance [1]. In addition, A CHAID analysis of interaction and homogeneity of the entire cohort demonstrates that education is the strongest predictor of uptake, followed by age, and occupational status. In the nested cohort the strongest predictor was occupational status, followed by age. The CHAID furthermore group occupational status in self-employed and social-welfare recipients or employed and people on unemployment benefits. This grouping was not seen in a comparable study in a neighbouring municipality showing similar uptake among employed and self-employed hand lowest uptake among social welfare recipients [29] .The grouping of employed and short-term unemployed may be due to the fact that unemployment benefits only lasts 2 years and that these people are formerly employed and likely and to re-enter the labor market within a short time-span. The grouping of the self-employed and people on social welfare is not as obvious but may be due to an unusually low attendance among a small group of self-employed (*N* = 28, 2% of the nested cohort), and not so much that these groups share reasons for non-attendance.

The low uptake of preventive studies and studies in general from residents of deprived settlements and ethnic minorities is well established [1, 30]. In the ‘Your Life – Your Health’ program social welfare recipients and people with non-Danish origin represent more than half of the nested cohort. An evaluation of the Danish national health survey shows that the odds of responding to a health survey is three times higher for people with Danish origin compared to people with non-Danish origin (descendants and immigrants) [31]. Even though we see a significantly higher attendance rate among people of Danish origin, the CHAID analysis indicates that educational attainment and occupational status, rather than country of origin, are the strongest predictors of attendance. In turn, this suggests that the association between attendance and country of origin may be mediated by educational attainment and occupational status. In light of this, efforts to improve the uptake and eliminate the differences we see between attenders and non-attenders should thus target people with low educational level as well as the unemployed. Studies from The Netherlands and Scotland suggest that a face-to-face community-based approach, engaging peers and community leaders in outreach work can increase the attendance at health checks in socially deprived settlements [32, 33].

### Targeted approach or mass screening

Two main assumptions underpin the simulation comparing the effects of targeted versus mass screening in Scotland: 1) The uptake of the two approaches is equal, and 2) a targeted approach will reach a higher proportion of people at risk (not the worried well or people already in treatment) the higher the social deprivation of the area [12].

The veracity of the first assumption of at least comparable uptake of mass screening and targeting socially deprived areas, is brought into question by the five-point discrepancy (30 and 25%) in uptake of the ‘Your Life – Your Health’ program. A similar study in a neighbouring municipality to Aarhus aimed at the general population had an overall uptake of 55%, but only 37% in people with low income and low educational level [29]. The simulation from Scotland shows a NNS of 16 in mass screening to identify one individual at high risk for CVD and six in targeted screening in socially deprived areas – in other words, a NNS that is 2.7 times lower when targeting socially deprived areas than any mass screening of the general population [12]. An uptake of 30 and 25% in the social housing sector in general and in a particularly socially derived settlement respectively, equals an attendance rate of between 81% and 67 in the general population of people residing in the social housing sector in general and in a socially deprived settlement when taking the proportion of people at-risk into consideration. Even though these figures favor the targeted approach used in the ‘Your Life – Your Health’ program they should be interpreted with caution as the reported attendance rate does not take into account potential confounding variables. These may include differences in context and characteristics of attendees and non-attendees in the two programs. A meta-analysis of the attendance rates in the two programs may be necessary to ascertain whether the targeted approach is more effective than mass screening regardless of varying NNSs.

The second assumption is that the program targets the at-risk population. A Dutch study on cardiovascular screening in general practice in a low socio-economic area suggested that the majority of people in this particular area could be classified as being at risk. Specifically, 60% of men and women above age 50 and 55, respectively, were not prescribed medication for hypertension or hypercholesterolemia despite a SCORE risk greater than 5% [34]. Using a broader definition of medical treatment, 40 and 39% in the entire cohort and the nested cohort, respectively, were not prescribed medical treatment. Further, people who were prescribed medical treatment were significantly less likely to attend a health check in the entire cohort of the ‘Your Life – Your Health’ program. In contrast we see no association between being prescribed medical treatment and attendance in the nested cohort. Thus, it would seem that the uptake of the ‘Your Life – Your Health’ program was comparable for people who had not been prescribed medical treatment and people who had. With the available information we are not able to see whether the program reaches people at risk, that is, attenders not being prescribed medicine with e.g. a SCORE above 5%, compared to the worried well, that is, attenders with e.g. a SCORE below 5% and a healthy lifestyle. In the ‘Your Life – Your Health’ program, increased attendance at health checks was associated with high income, high educational attainment, and employment, with the latter two variables representing the strongest predictors of attendance. Even though the settlements included in the ‘Your Life – Your Health’ program are more deprived and comprise a population that differs from the general population (Table 1), more health data on the attendees is required to establish whether attendees belong to the at-risk population or the worried well.

A third assumption not mentioned in the simulation from Scotland relates to the idea that an intervention should reach people who are not only at risk but who would not otherwise consult for cardiovascular screening (compliers). If the intervention merely taps populations of people who consult for cardiovascular screening both as part of usual care and when provided the opportunity through an intervention as the one described here (always-users), any effect will likely be negligible as these individuals are already relatively vigilant in attending health checks. In other words, an intervention that fails to reach compliers will in all likelihood contribute little or nothing to population health beyond that of standard care [35]. In the ‘Your Life – Your Health’ program being registered with an administrative code for a preventive consultation at the GP, or with attendance frequency at the GP had no impact on attendance. Of the two variables, being registered with an administrative code for a preventive consultation at the GP is probably the best proxy for a health check. The code indicates consultations specifically designed for encouraging behavior change or preventive medical treatment. The downside of the administrative code for a preventive consultation is that it is used to denote both primary and secondary prevention. Thus, the mere presence of this administrative code in a patient file, says nothing about whether the patient is at risk of disease, or if he/she is already ill. In light of this, data on biomarkers such as blood-pressure, cholesterol, or blood-sugar levels are required to establish whether intervention effects will be due to the fact, that the intervention reaches compliers and not always-users.

### Strengths and limitations

The main strengths of the present study are the high validity of the registries from Statistics Denmark and the random sampling of a complete cohort of residents from the social housing sector in the municipality of Aarhus [17]. The registries on income and occupational status, and the prescription registry is of very high quality with few missings and as such not prone to information bias [19, 20, 22]. The relatively large proportion of missing data on educational attainment is most likely due to yet-to-be-registered information on immigrants [21]. Nonetheless, missing data could possibly lead to information bias in the association between educational attainment and attendance. This, however, does not seem to be the case (Additional file 2). Administrative codes from the primary care sector have not been scrutinized in validation studies and may be prone to misclassification [18]. Contacts such as the 0101 code are registered using a personal health insurance card and is most likely both complete and valid. Contrary, service codes such as 0120 are registered by the GP in addition to each consultation and should be used with care, as they may be prone to misclassification. An evaluation of the administrative code for preventive consultations show large differences between GPs in the use of the code [36].

Another strength is the combination of DAG and CHAID analyses to establish a both theory-driven and data-driven analytical approach. DAGs establishes associations based on theoretical understanding of predictors of uptake of health checks while CHAIDs pinpoints the strongest predictors of attendance based on the available data.

The reliability of the analyses may have been slightly offset by possible residual confounding due to a complex causal model and unavailability of a number of exposures. The causal model is rather complex with a large number of exposures and causal paths. This may give rise to residual confounding due to incorrect causal paths and unavailability of certain confounders - for instance, information on health-risk behaviors, and cognitive variables such as health beliefs, and health literacy. Some of the exposures included in the present analyses – such as age, sex, country of origin, and educational attainment – are time-independent, early-in-life exposures with a rather straightforward analysis. Others, such as occupational status, income, cohabitation, medical treatment, and health-seeking behavior are time-dependent and challenged by reverse causation, collider bias, and residual confounding. However, most of the time-dependant variables are fairly stable over time, limiting challenges with reverse causation. Though some collider stratification bias and residual confounding is likely, the effects of these biases are negligent due to the strong predictive value of educational attainment, occupational status and age as demonstrated in the CHAID analysis.

A final limitation to the study is the high proportion of people of non-Danish origin and the information leaflet and questionnaire being available in Danish only. Firstly, even though the invitation was provided in four other languages than Danish, the Danish-only information leaflet and questionnaire may have had a significant impact on the overall attendance to the health checks. Secondly, even though the consent to use the data from the health check for research purposes was placed at the very end of the questionnaire, someone with poor reading and writing skills, and no help from a peer, could have provided consent without a proper understanding of what they consented to. The same might have been the case when the questionnaire was filled-in at the health centre together with a native Danish speaking health professional without the presence of an authorized interpreter.

## Conclusion

Attendance in the ‘Your Life – Your Health’ program was generally low and even lower in a sub-group living in an exceptionally deprived social housing area. Attendance was higher among people of a higher socio-economic status, females, people of Danish origin and increased with age. Education was the strongest predictor of attendance in the ‘Your Life – Your Health’ program. In an exceptionally deprived social housing area occupation was the strongest predictor. Attendance showed no association with income. These findings should be taken into consideration when analysing and interpreting the overall study effects. Moreover, the results suggest that a targeted approach in the social housing sector could be more effective than a mass screening approach. However, more information is required to make such assertion definitive.

## Additional files

Additional file 1: Direct Acyclic Graph (DAC) of determinants of attendance to health checks. (PDF 432 kb)

Additional file 2: Analysis of the impact of missings in the association between attendance and the variable educational attainment. (PDF 42 kb)

## Abbreviations

ATC: Anatomical Therapeutic Chemical Classification System
CHAID: Chi-squared automatic interaction detection
COPD: Chronic obstructive pulmonary disease
CVD: Cardiovascular disease
DAG: Direct acyclic graph
GP: General practitioner
IRR: Incidence rate ratio
NHS: National Health Services
NNS: Numbers needed to screen
OECD: Organisation for Economic Co-operation
OR: Odds ratio
SES: Socio-economic status
T2DM: Type-2 Diabetes Mellitus
UNESCO: United Nations Educational, Scientific and Cultural Organisation
