# Supplementary material for: Uptake of health checks by residents from the Danish social housing sector – a register-based cross-sectional study of patient characteristics in the ‘Your Life – Your Health’ program
Source: BMC Public Health. 2018 May 2;18:585. doi: 10.1186/s12889-018-5506-6 (PMC5932768; doi:10.1186/s12889-018-5506-6)
Supplement: Supplementary file 1 — Direct Acyclic Graph (DAC) of determinants of attendance to health checks. (PDF 432 kb) [file 12889_2018_5506_MOESM1_ESM.pdf]

Additional file 1 – Direct Acyclic Graph (DAC) of determinants of attendance to health checks

Dryden et al performed a systematic review of what we know about who does and does not attend general health checks from the patients point of view (1). According to Dryden et al, attendance is higher with older age, female gender, being married and of higher socio-demographic, white ethnic background and frequent attendance to general practice. Contrary, people with health risk behaviour such as smokers, obese, people with unhealthy diet, heavy drinkers, and people with raised biomarkers such as high blood pressure, cholesterol and blood sugar, are less likely to attend.   The higher attendance among married was explained by the fact that many, especially men, are encouraged by the partner to attend the health check. Socio-demographic factors include income, educational attainment and occupation and should be viewed as independent factors. Frequent attenders to general practice are more likely to attend general health checks. However, Dryden et al describe the association as being complex, that is, people use the same reasons for attendance and non-attendance. This is supported by a review of frequent attendance to general practice care where increasing age, being female, lack of social support, physiological disease and psychological distress are shown to predict frequent attendance (2).

Dryden et al also describe an association between social cognitive factors and attendance, though the relation is described as not being straightforward. As for frequent attendance,

people seem to use the same reasons for both attendance and non-attendance. Despite the complex relation, non-attenders show low self-efficacy, low control of health, and are less likely to belief in the effects of health checks. In a systematic review of qualitative studies on the perceptions and experiences of chronic disease programmes, Shaw et al provide a deeper look into the cognitive determinants that triggers uptake and effect (3). They present a conceptual model demonstrating that the cues to action, or inaction, to take up or respond to an intervention is determined by a combination of the patient's knowledge, beliefs, role and identity, followed by support to the prevention programmes. This includes the wider societal influences of the understanding of the role of, and trust in the state, and in more contextual influences made up of the health care professionals and health care sector support to preventive programmes.

Environmental factors such as neighbourhood deprivation seems to be associated with uptake while urban/rural residential area on the other hand seems unrelated to uptake (4). A number of papers report an increased uptake among people living in more deprived areas in the NHS Health Check (5,6). Other papers reporting from the NHS Health Check report higher uptake in less deprived areas (7–9). In a Danish setting, Bender et al show that low social capital and low social neighbourhood deprivation are associated with non-attendance to health checks, and that social capital is a likely mediator of the association between local neighbourhood deprivation and attendance to health checks (10,11).

When it comes to health care characteristics, several papers describe large differences in attendance between general practitioners (GPs), but the underlying causes of the association

is not described further (5,7,8,12). Distance to the GP does not seem to be associated with attendance to health checks (7). Practice size seems to matter, though the results point to higher attendance in both small and larger practices suggesting that practise size could be a mediator of some other underlying factor (6,13). According to Shaw, information on the role of health care professionals and uptake of preventive health checks is lacking (3).

Based on the aforementioned evidence a DAG was developed (figure 1).

Figure 1 - DAG depicting the determinants of attendance to health checks.

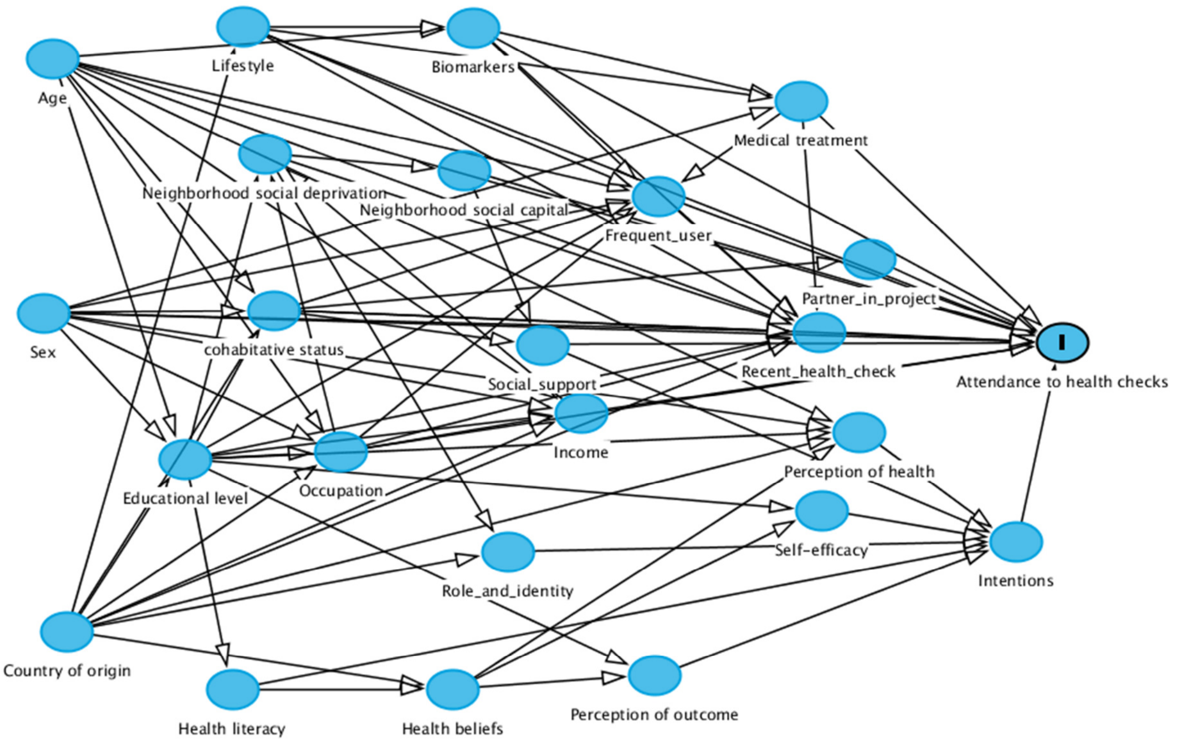

Can also be found at www.dagitty.net/mtRW08f

References

1. Dryden R, Williams B, McCowan C, Themessl-Huber M. What do we know about who does and does not attend general health checks? Findings from a narrative scoping review. BMC Public Health. 2012;12:723.

2. Vedsted P, Christensen MB. Frequent attenders in general practice care: a literature review with special reference to methodological considerations. Public Health. 2005 Feb;119(2):118–37.

3. Shaw RL, Holland C, Pattison HM, Cooke R. Patients' perceptions and experiences of cardiovascular disease and diabetes prevention programmes: A systematic review and framework synthesis using the Theoretical Domains Framework. Soc Sci Med 1982. 2016 May;156:192–203.

4. Hoebel J, Starker A, Jordan S, Richter M, Lampert T. Determinants of health check attendance in adults: findings from the cross-sectional German Health Update (GEDA) study. BMC Public Health. 2014;14(1):913.

5. Artac M, Dalton ARH, Babu H, Bates S, Millett C, Majeed A. Primary care and population factors associated with NHS Health Check coverage: a national cross-sectional study. J Public Health Oxf Engl. 2013 Sep;35(3):431–9.

6. Artac M, Dalton ARH, Majeed A, Car J, Huckvale K, Millett C. Uptake of the NHS Health Check programme in an urban setting. Fam Pract. 2013 Aug;30(4):426–35.

7. Gidlow C, Ellis N, Randall J, Cowap L, Smith G, Iqbal Z, et al. Method of invitation and geographical proximity as predictors of NHS Health Check uptake. J Public Health Oxf Engl. 2014 Nov 26;

8. Cochrane T, Gidlow CJ, Kumar J, Mawby Y, Iqbal Z, Chambers RM. Cross-sectional review of the response and treatment uptake from the NHS Health Checks programme in Stoke on Trent. J Public Health Oxf Engl. 2013 Mar;35(1):92–8.

9. Cook EJ, Sharp C, Randhawa G, Guppy A, Gangotra R, Cox J. Who uses NHS health checks? Investigating the impact of ethnicity and gender and method of invitation on uptake of NHS health checks. Int J Equity Health. 2016 Jan 20;15:13.

10. Bender AM, Kawachi I, Jørgensen T, Pisinger C. Neighborhood deprivation is strongly associated with participation in a population-based health check. PloS One. 2015;10(6):e0129819.

11. Bender AM, Kawachi I, Jørgensen T, Pisinger C. Neighborhood social capital is associated with participation in health checks of a general population: a multilevel analysis of a

population-based lifestyle intervention- the Inter99 study. BMC Public Health. 2015;15:694.

12. Lang S-J, Abel GA, Mant J, Mullis R. Impact of socioeconomic deprivation on screening for cardiovascular disease risk in a primary prevention population: a cross-sectional study. BMJ Open [Internet]. 2016 Mar 21 [cited 2017 Feb 15];6(3). Available from: http://www.ncbi.nlm.nih.gov/pmc/articles/PMC4809080/

13. Dalton ARH, Bottle A, Okoro C, Majeed A, Millett C. Uptake of the NHS Health Checks programme in a deprived, culturally diverse setting: cross-sectional study. J Public Health. 2011 Sep 1;33(3):422–9.
