# Supplementary material for: Uptake of health checks by residents from the Danish social housing sector – a register-based cross-sectional study of patient characteristics in the ‘Your Life – Your Health’ program
Source: BMC Public Health. 2018 May 2;18:585. doi: 10.1186/s12889-018-5506-6 (PMC5932768; doi:10.1186/s12889-018-5506-6)
Supplement: Supplementary file 2 — Analysis of the impact of missings in the association between attendance and the variable educational attainment. (PDF 42 kb) [file 12889_2018_5506_MOESM2_ESM.pdf]

Additional file 2 – Analysis of the impact of missings in the association between attendance and the variable educational attainment

**Missings not incuded in the analysis**				
	Entire cohort (n=6217)		Nested cohort (n=1227)	
	Model 2 (age, sex) IRR(95% CI) p-value	Model 3 (minimally adjusted)	Model 2 (age, sex)	Model 3 (minimally adjusted)
< 10 år	ref	ref	ref	ref
10 – 15 år	1.37 (1.25;1.51) 0.000	1.36 (1.24;1.50) 0.000	1.47 (1.16;1.85) 0.001	1.45 (1.15;1.83) 0.002
> 15 år	1.57 (1.42;1.74) 0.000	1.56 (1.41;1.73) 0.000	1.70 (1.32;2.19) 0.000	1.67 (1.30;2.15) 0.000

**Missings included in the analysis as a category**				
	Entire cohort (n=6649)		Nested cohort	
	Model 2 (age, sex) IRR(95% CI) p-value	Model 3 (minimally adjusted)	Model 2 (age, sex)	Model 3 (minimally adjusted)
< 10 år	ref	ref	ref	ref
10 – 15 år	1.37(1.25;1.51) 0.000	1.37(1.24;1.50) 0.000	1.48(1.17;1.87) 0.001	1.46(1.16;1.85) 0.001
> 15 år	1.57(1.42;1.74) 0.000	1.56(1.40;1.73) 0.000	1.71(1.32;2.20) 0.000	1.68(1.30;2.16) 0.000
Missings	1.02(0.85;1.24) 0.808	1.06(0.87;1.28) 0.582	1.10(0.79;1.53) 0.568	1.15(0.82;1.62) 0.415

**Missings coded as educational attainment < 10 years**				
	Entire cohort (n=6649)		Nested cohort (n=1407)	
	Model 2 (age, sex) IRR(95% CI) p-value	Model 3 (minimally adjusted)	Model 2 (age, sex)	Model 3 (minimally adjusted)
< 10 år	ref	ref	ref	ref
10 – 15 år	1.37(1.25;1.49) 0.000	1.35(1.23;1.48) 0.000	1.43(1.16;1.76) 0.001	1.40(1.14;1.72) 0.002
> 15 år	1.56(1.41;1.72) 0.000	1.54(1.40;1.70) 0.000	1.65(1.31;2.08) 0.000	1.61(1.28;2.02) 0.000

**Missings coded as educational attainment between 10 and 15 years**				
	Entire cohort (n=6649)		Nested cohort (n=1407)	
	Model 2 (age, sex) IRR(95% CI) p-value	Model 3 (minimally adjusted)	Model 2 (age, sex)	Model 3 (minimally adjusted)
< 10 år	ref	ref	ref	ref
10 – 15 år	1.33(1.21;1.46) 0.000	1.33(1.21;1.46) 0.000	1.38(1.10;1.73) 0.005	1.39(1.11;1.74) 0.004
> 15 år	1.57(1.42;1.74) 0.000	1.56(1.41;1.73) 0.000	1.71(1.32;2.20) 0.000	1.68(1.30;2.16) 0.000

**Missings coded as educational attainment ≥ 15 years**				
	Entire cohort (n=6649)		Nested cohort (n=1407)	
	Model 2 (age, sex) IRR(95% CI) p-value	Model 3 (minimally adjusted)	Model 2 (age, sex)	Model 3 (minimally adjusted)
< 10 år	ref	ref	ref	ref
10 – 15 år	1.37(1.25;1.51) 0.000	1.36(1.24;1.50) 0.000	1.48(1.17;1.86) 0.001	1.46(1.16;1.84) 0.001
> 15 år	1.46(1.32;1.61) 0.000	1.46(1.32;1.62) 0.000	1.47(1.16;1.86) 0.001	1.49(1.17;1.90) 0.001
